# Androgen Excess Disorders in Women: The Severe Insulin-Resistant Hyperandrogenic Syndrome, HAIR-AN

**DOI:** 10.1100/tsw.2006.23

**Published:** 2006-01-24

**Authors:** Kristin M. Rager, Hatim A. Omar

**Affiliations:** Department of Pediatrics, University of Kentucky College of Medicine, Lexington, KY 40536, USA

**Keywords:** hyperandrogenism, acanthosis nigricans, insulin resistance, United States

## Abstract

HAIR-AN syndrome (hyperandrogenism, insulin resistance, acanthosis nigricans) is a subset of the polycystic ovary syndrome, where the patients demonstrate severe insulin resistance. It is theorized that both genetic and environmental factors, such as obesity, give rise to the development of HAIR-AN. Diagnosis is primarily clinical, with laboratory values lending further support. Treatment is aimed at decreasing insulin resistance, regulating ovulation, and decreasing acne, acanthosis nigricans, and hirsutism.

## INTRODUCTION

HAIR-AN syndrome, which is characterized by hyperandrogenism, insulin resistance, and acanthosis nigricans, is a subphenotype of the polycystic ovary syndrome. This review will discuss the clinical presentation, genetic and environmental contributors, diagnosis, and interventions for treatment of this syndrome.

## BACKGROUND

The polycystic ovary syndrome (PCOS) is a disease of infrequent or absent ovulation and hyperandrogenism. HAIR-AN syndrome is a specific subtype of PCOS characterized by the presence of severe insulin resistance[1,2]. Approximately 5–10% of females with hyperandrogenism have HAIR-AN[3,4] and up to 40% of adolescent patients who present with irregular periods may have HAIR-AN[5]. Most women with HAIR-AN will have clinical symptoms in adolescence; however, diagnosis is often delayed until adulthood[6,7].

## Clinical Presentation

Clinically, hyperandrogenism associated with HAIR-AN may give rise to acne and slowly progressive hirsutism. Virilization, which includes temporal balding, voice deepening, rapidly progressive hirsutism, and clitoromegaly generally suggest more significant androgen exposure and warrant further evaluation[1,3]. Hirsutism is defined by an increase in the distribution and quantity of terminal hairs (>0.5 cm in length, coarse, usually pigmented) representing a Ferriman and Gallwey score of ≥8[8].

Clinical findings related to insulin resistance are body mass index (BMI) >27 kg/m$^2$, waist to hip ratio >0.85, waist >100 cm, numerous skin tags (achrochordons), and acanthosis nigricans[9]. Acanthosis nigricans is velvety, verrucous, hyperpigmented skin found most frequently on the back of the neck, axillae, and in other skin fold areas (see Fig. 1), and is a known indicator of underlying insulin resistance and decreased insulin sensitivity. Undiagnosed and untreated insulin resistance is linked to long-term sequelae, such as coronary artery disease, hyperlipidemia, and Type II diabetes[10].

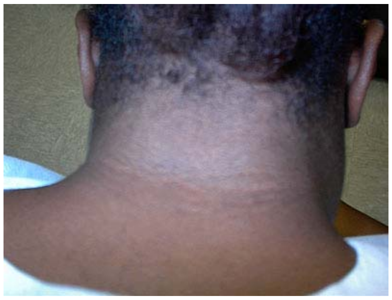

**FIGURE 1.**

## Insulin Resistance/Hyperinsulinemia

Hyperinsulinemia has been a known component of PCOS since at least 1984[11]. As obesity and body fat distribution have known effects on glucose metabolism, this contributes to the insulin resistance in HAIR-AN[12,13] in obese women. Certainly not all women with HAIR-AN are obese, and when the above variables are controlled for, insulin resistance is still present[14,15]. In addition, obesity and PCOS have been found to have a synergistic effect on increasing insulin resistance[14,16]. As insulin resistance increases, pancreatic β cells increase insulin production to compensate. However, in women with HAIR-AN, insulin response to glucose intake is attenuated[17] and defects in β cell function have been identified[18,19]. Genetic defects, such as mutations of the tyrosine kinase domain of the insulin receptor gene, have also been suggested in the pathogenesis of HAIR-AN[20,21].

## Hyperandrogenism

The prolonged, markedly elevated, insulin levels, which are associated with resistance and decreased sensitivity, may stimulate the ovary to generate androgens[1,20]. It is theorized that these high levels of insulin cross-react with insulin-like growth factors, therefore, directly stimulating the overproduction of ovarian androgens[1,10]. This gives rise to the hyperandrogenism associated with HAIR-AN.

## Diagnosis

There is no consistently used "gold standard" of diagnostic testing for HAIR-AN. Diagnosis is often based on the clinical findings, i.e., evidence of hyperandrogenism (acne, hirsutism) and evidence of insulin resitance (acanthosis nigricans)[5], but on the other hand, many women with insulin resistance do not have acanthosis nigricans[9,20]. Multiple laboratory tests have been posited as tools in the diagnosis of HAIR-AN, including fasting insulin levels, fasting glucose-to-insulin ratio, glucose challenge testing, and the euglycemic hyperinsulinemic clamp. These lab values can be helpful; however, the only measure that is both sensitive and specific is the euglycemic hyperinsulinemic clamp. Regrettably, this test is both impractically complex and time consuming, and too expensive for the clinic setting[20]. Currently, there is no solid consensus as to what testing is most beneficial in the evaluation of patients with possible HAIR-AN.

## Treatment

Likewise, there is no consensus on the "standard of care" for the treatment of HAIR-AN syndrome. Traditional treatments have included weight loss, oral contraceptive pills, and antiandrogens. Debate continues regarding treatment with insulin-sensitizing agents, such as metformin.

BMI is known to be directly proportional to insulin resistance[22]. Multiple studies have demonstrated that obese women with HAIR-AN can have decreased androgens and return of regular ovulation with weight loss[23,24,25]. As obesity has reached epidemic proportions in the U.S., achieving weight loss in patients, although not often easy, can have health benefits beyond those directly attributable to HAIR-AN. There is evidence that weight loss is more easily achieved after the hyperandrogenism and hyperinsulinemia have improved[26].

Oral contraceptive pills (OCP) are often used to improve menstrual cycle regularity, improve acne, and decrease hirsutism, and possess the added benefit of prevention of unintended pregnancy[27]. OCP may suppress the hypothalamic-pituitary-ovarian axis, therefore lowering ovarian steroid/androgen production[1]. There is no evidence that OCP improve insulin resistance or sensitivity. Some evidence suggests that OCP should not be used in patients with HAIR-AN, as they might decrease insulin sensitivity[28]. Recently, Yasmin® (Berlex), an OCP with drospirenone, a spironolactone analog, has been introduced that may be used in the treatment of the hyperandrogenism associated with HAIR-AN[29].

Antiandrogens, often in conjunction with OCP, may be utilized to decrease acne and hirsutism. These include spironolactone, GnRH agonists, and flutamide. Spironolactone frequently is used in conjunction with OCP to target hirsutism. Antiandrogens should not be used in sexually active women who are not on contraception, as they can have adverse fetal effects.

Treatments aimed at directly increasing insulin sensitivity are the topic of much debate. Insulin sensitizers that are currently available in the U.S. include metformin, rosiglitazone, and pioglitazone (troglitazone is no longer available). Metformin is approved by the FDA for treatment of Type II diabetes mellitus (but not for insulin resistance), and in addition to enhancing peripheral glucose uptake, metformin increases insulin sensitivity. Metformin is the only insulin sensitizer that is approved for use in those patients 10–16 years old; however, recent data suggest that rosiglitazone may be beneficial in pediatric patients[30]. One of the most important clinical outcomes is the improvement in menstrual regularity/ovulation. Randomized, placebo-controlled trials of metformin and rosiglitazone in obese patients with PCOS have demonstrated ovulation rates of 30–42%[31,32,33]. In those with PCOS and severe insulin resistance, ovulation rates have reached 92%[34]. Insulin-sensitizing agents logically are more likely to induce ovulation in women with higher levels of insulin resistance[32,33,34]. Therefore, insulin-sensitizing agents are most useful for use in the HAIR-AN subset of PCOS.

Insulin-sensitizing agents also improve hyperandrogenism. Metformin and pioglitazone have been shown to decrease serum androgens[31,35,36]. The decreased serum androgen levels are likely a direct consequence of decreased serum insulin levels.

Two small studies have been carried out in adolescents using metformin and dietary intervention. Both revealed weight loss and resumption of menses; however, the metformin effects were confounded by the dietary interventions[37,38]. In addition to the potential for metabolic acidosis and liver damage, metformin gives rise to gastrointestinal side effects which patients often dislike.

## CONCLUSIONS

HAIR-AN syndrome is a disease that manifests in adolescence, yet may have long-reaching consequences into adulthood. It is important to recognize the symptoms as they present and to treat accordingly. Although HAIR-AN syndrome was first described over 30 years ago and many advances since been made, there are still areas which require continuing research: (1) continued insight into the genetic causes and their relationships with environmental influences, (2) continued development and evaluation of programs targeting the obesity epidemic, (3) development of more practical sensitive and specific laboratory diagnostic methods, (4) further investigation of treatment modalities, specifically of the insulin-sensitizing agents, (5) further investigation of the insulin-sensitizing agents in adolescent populations, and (6) studies evaluating long-term impact of treatment with insulin-sensitizing agents on the development of Type II diabetes and cardiovascular disease in patients with HAIR-AN.

## KEY POINTS

- HAIR-AN is a subphenotype of polycystic ovary syndrome (PCOS), which differs by the presence of insulin resistance.
- Acanthosis nigricans, acne, hirsutism, and menstrual irregularity should alert the provider to the possibility their patient has HAIR-AN.
- Easily obtained laboratory values may assist with the diagnosis of HAIR-AN, but are not diagnostic in themselves.
- Weight loss, oral contraceptive pills (OCP) use, and antiandrogens may be used in the treatment of HAIR-AN.
- Insulin-sensitizing agents may also be used; however, their use is currently controversial based on potential side effects and lack of solid long-term data.

## BIOSKETCHES

**Kristin M. Rager**, **MD**, Assistant Professor, Section of Adolescent Medicine, Department of Pediatrics, University of Kentucky College of Medicine, Lexington. E-mail: Rager@uky.edu

**Hatim A. Omar, M**D is Professor of Pediatrics and Obstetrics/Gynecology and Director of Adolescent Medicine and Young Parent programs at the Kentucky Clinic, University of Kentucky, Lexington. Dr. Omar has numerous publications in child health, adolescent health, medicine, obstetrics, and gynecology. E-mail: haomar2@uky.edu
